# Characterization of the Polysaccharide and Protein Composition of *Neurospora crassa* Conidium, Perithecium, and Ascospore Cell Walls

**DOI:** 10.3390/jof12090657

**Published:** 2026-09-02

**Authors:** Protyusha Dey, Ian Black, Christian Heiss, Liyanage Devthilini Fernando, Parastoo Azadi, Piotr Shtyk, Stephen J. Free

**Affiliations:** 1 Department of Biological Sciences, SUNY University at Buffalo, Buffalo, NY 14260, USA; protyusha.dey@roswellpark.org (P.D.);; 2 Complex Carbohydrate Research Center, University of Georgia, Athens, GA 30602, USA

**Keywords:** fungal cell wall, ascospore, conidia, perithecia, Neurospora, glucan, chitin, melanin

## Abstract

We carried out carbohydrate composition and carbohydrate linkage analyses on *Neurospora crassa* cell walls from vegetative hyphae, conidia (asexual spores), perithecia (female mating structures), and ascospores (sexual spores). We show that the composition and structure of the cell wall undergo dramatic changes during the *N. crassa* life cycle. All the cell walls contain β-1,3-glucan and mixed β-1,3-/β-1,4-glucan (lichenin). The conidia cell walls and perithecia cell walls also contain α-1,3-glucan. β-1,3-glucan was the most abundant polysaccharide in the vegetative hyphae cell walls and mixed β-1,3-/β-1,4-glucan was the most abundant polysaccharide in the perithecia and ascospore cell walls. Chitin was a major polysaccharide in the ascospore cell walls. Ascospore and perithecia cell walls also contained melanin as a structural element. We conclude that the composition and structure of the cell wall changes dramatically as cells proceed through the various stages of the *N. crassa* life cycle.

## 1. Introduction

The fungal cell wall is a cross-linked matrix of glucans, chitin, and glycoproteins that is essential for the survival, growth, and development of fungi. It protects the fungal cell from desiccation, heat, and other environmental stresses and is an important determinant of cell morphology [1,2,3]. Since the fungal cell wall structure and the enzymes involved in cross-linking the wall together are unique to fungi, the cell wall has been a major target for the development of antifungal agents. Most of the studies on the fungal cell wall have been focused on cell walls produced during vegetative growth, with a very limited number of studies on the composition of cell walls produced at other stages of fungal life cycles. The polysaccharides present in cell walls of vegetative hyphae have been shown to include β-1,3-glucans, α-1,3-glucans, mixed β-1,3-/β1,4-glucans, β-1,6-glucans, and chitin [1,2,4]. A number of different cell wall glycoproteins have been identified and functions for some of these glycoproteins have been characterized. These functions include roles in the formation and cross-linking of the cell wall polysaccharide matrix, adhesion, and other functions. However, only a few studies have looked at how the fungal cell wall changes as fungi undergo developmental processes and generate other cell types [5,6,7,8,9].

To determine how the *N. crassa* cell wall changes during the different stages of the Neurospora life cycle, we have isolated and characterized cell walls from four different major cell types. We analyzed the sugar composition, the sugar linkages, and the glycoprotein contents of cell walls from vegetative hyphae, conidia (asexual spores), perithecia (the female mating structure), and ascospores (sexual spores). The analyses show that each of these cell walls differs markedly from the others in the polysaccharides present, the abundance of the different types of polysaccharides, and in the glycoproteins present. β-1,3-glucan and a mixed β-1,3-/β-1,4-glucan (lichenin) were found to be present in the four types of cell walls. β-1,3-glucan was the most abundant glucan in vegetative cell walls. Mixed β-1,3-/β-1,4-glucan (lichenin) was the most abundant glucan in the other cell walls. Chitin was found to be a major polysaccharide within the ascospore cell walls and a minor component in the other cell walls. A previous study demonstrated the conidia cell wall contains α-1,3-glucan [10]. We show that α-1,3-glucan was also expressed in the perithecia. We did not find evidence for β-1,6-glucan within any of the cell walls examined. The absence of β-1,6-glucan has been noted in our previous analyses of *N. crassa* vegetative cell walls. The cell walls differed from each other in the amount of glycoprotein present. The ascospore and perithecia cell walls contain significant amounts of melanin. In summary, each of the four cell walls examined has a unique repertoire of polysaccharides and there were some dramatic differences between the different cell walls. Our results suggest that the organization of ascospore cell walls, with a large amount of chitin, glycoprotein, and melanin, provides the formidable cell wall structure characteristic of *N. crassa* ascospores, which are able to survive for decades in the soil and become activated following a forest fire.

## 2. Materials and Methods

### 2.1. Strains and Growth Conditions

The wildtype isolates of *N. crassa* (OR23 *mt-A* and ORS *mt-a*) were obtained from the Fungal Genetics Stock Center (Manhattan, KS, USA) and used for cell wall analyses. The cells were routinely maintained in Vogel’s 2% sucrose agar medium [11].

### 2.2. Mating Experiments

To follow the expression of the *ags-1* (α-1,3-glucan synthase) gene, a strain with the *ags-1* promoter driving expression of red fluorescent protein (dimeric RFP) was used as a female in mating experiments [10]. Mating experiments were carried out on 60 mm circles of Whatman 3MM chromatography paper in Petri dishes with 15 mL of synthetic crossing medium [11]. The paper was inoculated with the protoperithecia partner (female) and the fungus was allowed to grow at room temperature in the dark for 5 days to allow for the development of protoperithecia. A suspension of conidia from a wildtype strain was then added to fertilize the protoperithecia and the plate was incubated at room temperature for 14 days. During the ensuing 14 days of perithecia maturation, developing perithecia were periodically picked from the Petri dishes with sterile tweezers. Perithecia squashes were prepared by placing several perithecia on a glass slide in approximately 20 µL of water, placing a cover slip over the perithecia, and squashing them by placing a soft eraser on the cover slip and pressing down on the cover slip to break the perithecia apart. The squashed perithecia were examined with a fluorescence microscope and RFP images were collected at 610–700 nm with excitation at 520–550 nm.

### 2.3. Isolation of Vegetative Cell Wall

Vegetative cell walls were obtained from wildtype strains growing in Vogel’s liquid medium supplemented with 2% sucrose [12,13,14,15,16]. The hyphae were grown in 50 mL of medium in 250 mL Erlenmeyer flasks at 30 °C in a shaker incubator (120 rpm) for 24 h. The cells were harvested on a Buchner funnel and ground to a fine powder in a mortar and pestle under liquid N_2_. An examination of the sample with light microscopy showed that over 99% of the hyphae were broken. The powdered cells were suspended in 10 mL of PBS and the cell walls collected by centrifugation at 6000× *g* for five minutes and washed with 10 mL of PBS. The cell walls were then suspended in 10 mL PBS with 1% SDS and the centrifuge tube with the cell walls was placed in a boiling water bath for 15 min. The cell walls were then collected by centrifugation and washed twice with PBS and three times with distilled water. The cell walls were then transferred to microfuge tubes and dried in a speed vac.

### 2.4. Isolation of Perithecia Cell Wall

To isolate perithecia cell walls, wildtype *mt-A* cells were inoculated on two 60 mm Whatman 3MM filters placed in Petri dishes in 15 mL of synthetic crossing medium without sugar supplementation (the filters provide the nutrients for *N. crassa* growth and protoperithecia formation). The Petri dishes were incubated in the dark at room temperature (22 °C) for five days to allow for protoperithecia to form. The protoperithecia were fertilized by the addition of a conidial suspension from the wildtype *mt-a* strain to the filters, and the plates were incubated for another 10 days at room temperature to allow perithecia to form and mature. Mature perithecia were harvested by picking perithecia with tweezers from beyond the edges of the filter paper being careful to avoid getting filter paper in the sample. The harvested perithecia were ground to a fine powder in a mortar and pestle under liquid N_2_ and the cell walls were prepared as described for the isolation of vegetative cell walls.

### 2.5. Isolation of Conidia Cell Wall

Conidia cell walls were obtained as previously described [17]. Conidia were harvested from 24 slants with 3 mL of Vogel’s 2% sucrose agar medium that had been inoculated with OR23 *mt-A* isolates and grown for 7 days at 22 °C. The conidia were harvested in distilled water, passed through four layers of cheesecloth to remove hyphae, and collected by centrifugation at 6000× *g*. The conidia were resuspended in 2 mL PBS, distributed into four Eppendorf Safe-Lock microfuge tubes containing 0.5 mm zirconium oxide beads, and disrupted in a Bullet Blender Storm (Next Advance, Averill Park, NY, USA) set at a speed setting of 10. The conidia were subjected to four 5 min cycles of disruption. The broken cell walls were removed from the microfuge tubes with a P200 Pipettor, collected by centrifugation (6000× *g* for 5 min), and washed with PBS. An examination of the conidia under the light microscopy demonstrated that over 99% of the conidia were disrupted. The conidia cell walls were then suspended in 10 mL PBS with 1% SDS, and the centrifuge tube was placed in a boiling water bath for 15 min to release proteins and polysaccharides not covalently attached to the cell walls. The cell walls were then washed twice with PBS and three times with distilled water. The cell walls were transferred to microfuge tubes and dried in a speed vac.

### 2.6. Isolation of Ascospore Cell Wall

Ten Petri dishes containing two 60 mm Whatman 3MM filters and 15 mL of synthetic crossing medium without sugar supplementation (the filters provide the nutrients for *N. crassa* growth and protoperithecia formation) were inoculated with wildtype *mt-A* conidia and incubated in the dark at room temperature (22 °C) for five days to allow for protoperithecia to form. The protoperithecia were fertilized by the addition of a conidial suspension from the wildtype *mt-a* strain to the filters, and the plates were incubated for another 21 days at room temperature to allow perithecia to mature and eject ascospores onto the lids of the Petri dishes. The ascospores were collected from the lids by adding 2 mL of distilled water to the upside-down lids and using a sterile spreader to wash the ascospores from the lid surface. The ascospores were removed from the lid by aspiration with a Pasteur pipet and collected by centrifugation (6000× *g* for 5 min). The ascospores were resuspended in PBS, transferred to Eppendorf Safe-Lock microfuge tubes with 0.5 mm zirconium oxide beads, and processed as described above for the isolation of conidia cell walls.

### 2.7. Composition Analysis by Trimethylsilyl (TMS) Derivatization and GC-MS

Glycosyl composition analyses were performed by forming per-O-trimethylsilyl (TMS) methyl glycosides after solubilizing insoluble polysaccharides by prior O-acetylation in ionic liquid as described by Black et al. [18]. For derivatization of the hydroxyl groups, 400 µg samples of cell wall were placed in a clean test tube fitted with a Teflon-lined screw cap. In addition, 400 μL of dry 1-ethyl-3-methylimidazolium acetate was added to the samples along with a stir bar. The samples were stirred for three days at room temperature to dissolve the samples. Then, 300 μL acetic anhydride and 50 μL N-methylimidazole were added to the samples and, after stirring for 10 min at room temperature, 2 mL water was added to quench the reaction. The acetylated samples were then extracted to dichloromethane (DCM) and washed with water five times. Finally, the DCM layers were dried and, after addition of 20 μg of inositol, the samples were lyophilized.

The lyophilized polysaccharide samples were subjected to methanolysis by the addition of 300 µL of 1 M methanolic HCl and incubation at 80 °C for 16 h to depolymerize the polysaccharides and convert the resulting monosaccharides into their corresponding methyl glycosides. Following cooling, the samples were dried under a stream of dry air. To remove residual acid, 200 µL of methanol was added and evaporated under dry air; this step was repeated three times.

Re-N-acetylation of amino sugars was performed by adding 200 µL of methanol, 100 µL of pyridine, and 100 µL of acetic anhydride to the dried samples followed by incubation at 50 °C for 1 h. The reaction mixtures were then dried completely under dry air. For derivatization of the hydroxyl groups, the samples were treated with 300 µL of Tri-Sil HTP reagent (Thermo Scientific, Waltham, MA, USA) at 80 °C for 20 min. The reaction mixtures were partially dried until approximately 50 µL remained, then hexane was added to precipitate the TMS byproducts. The supernatants were carefully transferred to a fresh tube and dried completely prior to GC-MS analysis.

The per-O-trimethylsilyl (TMS) methyl glycosides from the samples were dissolved in 100 μL of hexane and transferred to a GC vial. GC-MS analysis was performed on an Agilent 7890A GC (Agilent Technologies, Santa Clara, CA, USA) interfaced to a 5975C MSD using a Supelco Equity-1 fused silica capillary column (30 m × 0.25 mm ID). After 2 min at the initial temperature of 80 °C, the temperature was increased by 2°/min until 200 °C was reached, and then after a 2 min hold time, the temperature was raised at 3°/min until 250 °C was reached and held there for 5 min.

### 2.8. Linkage Analysis by Partially Methylated Alditol Acetate (PMAAs) and GC-MS Methylation

Briefly, 300 µL of DMSO was added to 500 µg aliquots of the cell wall samples and stirred for three days to dissolve the samples. In addition, 300 µL of NaOH suspension in DMSO was added to the samples and stirred for another 15 min. Then, 100 µL of iodomethane was added to the samples, which were then stirred for 25 min. The samples were then extracted with DCM, and the DCM layers were washed with water a total of 5 times. The DCM layers were then dried. The samples were lyophilized to ensure complete dryness.

For hydrolysis and anomeric group reduction of the methylated polysaccharides, 300 µL of 2 M trifluoroacetic acid (TFA) was added to the samples and the samples were heated for 2 h at 120 °C. The samples were then dried using isopropyl alcohol. To reduce the anomeric group, 300 µL of 10 mg/mL NaBH_4_ prepared in NH_4_OH was added to the samples and allowed to react overnight. The samples were then acidified by the addition of 100 µL of 9:1 methanol:acetic acid, and the samples were dried with a stream of dry air. The acid was then removed by 100 µL of methanol and the solvents evaporated with dry air; this step was repeated 3 more times.

To acetylate the samples, 250 µL pyridine and 250 µL acetic anhydride were added to the samples and allowed to react at 100 °C for 1 h. Then, the samples were concentrated to a volume of 100 µL. The samples were extracted with 2 mL of DCM, and the DCM layers were washed with 2 mL of water five times. After the last wash, the DCM layers were transferred to a new tube and dried. Finally, the samples were redissolved in 200 µL DCM and transferred to GC vial inserts.

The resulting partially methylated alditol acetates (PMAAs) were analyzed on an Agilent 7890A GC interfaced to a 5975C MSD; separation was performed on a Supelco 2331 fused silica capillary column (30 m 0.25 mm ID) in 10:1 split ratio with a temperature gradient as follows: after 1 min at the initial temperature of 60 °C, the temperature was increased by 4 °/min until 235 °C was reached, and then after a 2 min hold time, the temperature was raised by 3 °/min until 240 °C was reached and held there for 12 min. To detect amino sugar, the sample was analyzed on a Supelco Equity-1 fused silica capillary column (30 m × 0.25 mm ID).

## 3. Results

### 3.1. Comparison of Cell Wall Organization Across the N. crassa Life Cycle

To examine how the organization of the cell wall changes during the *N. crassa* life cycle, we purified cell walls from conidia (asexual spores), perithecia (female mating structure), and ascospores (sexual spores). A single sample for each of these three types of cell walls was used for analyses at the Complex Carbohydrate Research Center (Athens, GA). We identified the neutral sugars found in the cell walls from these different types of cells and compared their compositions to the neutral sugars from previous analyses of vegetative hyphae cell walls [12,13,14,15,16] (Table 1). We found there were clear differences in the cell wall compositions of these four cell types. Glucose was the major sugar present in all four cell types but there were significant differences in the amounts of glucose, mannose, and galactose. The mannose and galactose present in *N. crassa* cell walls are largely derived from the post-translational modification in cell wall glycoproteins [16], while glucose largely represents cell wall glucans. The composition analyses suggest that the conidia cell wall contains more glucan (94.1%) than the vegetative cell wall (83.2%) and contains less glycoprotein. Similarly, the perithecia cell wall contains more glucan (93.7%) and less glycoprotein than the vegetative cell wall. In contrast, the ascospore cell wall contains less glucan (77.6%) and more glycoprotein than the vegetative hyphae cell wall.

### 3.2. Linkage Analyses of Cell Wall Glucans Across the N. crassa Life Cycle

We also carried out an analysis of the glycosidic linkages present in the different types of cell walls (Table 2) and found that the linkage analyses largely corroborated the differences we had seen in cell wall glucan and glycoprotein content in the composition analysis (Table 1). In Table 2, the major cell wall glycosidic linkages found in the analyses (linkages representing 5% or more of the linkages seen in a sample) are shown in red. In the conidia cell wall sample, we found that 1,3-glucose linkages represented 75.7% of the linkages in the sample. We have previously shown that β-1,3-glucan is the major cell wall glucan in *N. crassa* cell walls [12,13,14,15,16]. We have also shown that the conidia wall contains α-1,3-linked glucan, which is lacking in vegetative hyphae [10]. The conidia cell walls also contained 1,4-linked glucoses (10.1%). Using antibody directed against lichenin (a mixed β-1,3/β-1,4-glucan consisting of a repeating β-1,4-glucose-β-1,4-glucose-β-1,3-glucose trisaccharide), we have shown that lichenin is a major cell wall glucan in *N. crassa* and we ascribe the 1,4-linked glucose in the vegetative hyphae and conidia to lichenin [13,19]. With 10.1% of the glucose in 1,4-linkages, we would calculate that approximately 15% of the conidia cell wall is lichenin and approximately 70% of the conidia cell wall consists of β-1,3-glucan and α-1,3-glucan. One interesting observation from our conidia cell wall linkage analysis is the abundance of 3,6-glucose (5.1% of the observed linkages), representing “branches” within 1,3-linked glucans. The 3,6-linked glucose represents glucose residues where one linear glucan has been attached to a second linear glucan to generate a cross-linked polysaccharide matrix [20]. Our data suggests that the glucans in the conidia cell wall may be more heavily cross-linked than in the other cell wall samples. The mannose and galactose found in *N. crassa* cell walls has been found to be largely derived from the post-translational modifications in glycoproteins [16,21]. The levels of mannose and galactose residues in the composition and linkage analyses suggest that conidia cell walls have approximately 1/3 the concentration of glycoproteins found in the vegetative hyphae cell walls. In this regard, it is worth noting that in our cell wall purification protocol, the cell walls were subjected to a heat step in the presence of 1% SDS to remove any proteins not covalently attached to the cell wall. This step would remove the outer hydrophobin protein layer that is non-covalently associated with the conidia cell wall [22].

The mole percentage for each of the sugar residues detected in a glucosyl-linkage analysis of cell wall samples from ascospores (Asc.), from perithecium (Per.), from three vegetative hyphae preparations, and from conidia is shown. The major cell wall polysaccharides detected, as defined by being greater than 5% of at least one cell wall sample, are highlighted in red. The sugar residues are identified by a t or a number followed by a—and by the sugar type. A t denotes a terminal sugar, and the number(s) denotes the linkage(s). The sugar residues are denoted as Galf for galactofuranose, Gal for galactose, Man for mannose, Glc for glucose, GlcNac for N-acetylglucosamine or glucosamine.

We have carried out several analyses of vegetative cell walls from hyphae grown in liquid medium [12,13,14,15,16] and the results from these analyses are included here for comparison. The major sugar linkages seen in vegetative cell walls are 1,3-glucose and 1,4-glucose. The 1,4-glucose comes from lichenin and represents about 16% of the linkages in the wall (Table 2). Given that lichenin is a repeating trisaccharide with two β-1,4 linkages and one β-1,3 linkage, we would estimate that lichenin represents approximately 24% of the cell wall and β-1,3-glucan represents approximately 51% of the cell wall. Based on the amounts of mannose and galactose, which come largely from cell wall glycoprotein, glycoproteins are much more abundant in the vegetative hyphae cell walls than in the conidia and perithecia cell walls (Table 1 and Table 2).

Perithecia are the highly melanized female mating structures in which meiosis occurs and ascospores are generated. While perithecia contain multiple cell types [23,24], the major portion of the perithecia consists of multiple layers of hyphae which form a protective layer (called a peridium) surrounding the developing ascospores. The outer layers of perithecia (outer peridium or outer perithecial wall cells) are derived from the haploid female parent. The inner layers of the peridium (inner peridium or centrum pseudoparenchymal cells) are derived from the more centrally located cells of the protoperithecia and could potentially include some dikaryotic cells with nuclei from both parents [25]. The outer peridium is heavily melanized. We harvested perithecia 10 days after fertilization and prepared cell wall samples for analysis. The data in Table 2 suggests that the highly melanized nature of the perithecia cell walls may have given some interference with the permethylation step in the linkage analysis (giving rise to products with “extra” linkages). An interference with the permethylation step in the linkage analysis would result in an underestimation of the amounts of 1,3- and mixed 1,3-/1,4 glucans in the cell wall samples. Nevertheless, the data demonstrates that the perithecia cell walls are clearly different from the other types of cell walls examined (Table 1 and Table 2). The compositional data shows that over 92% of the perithecia cell walls consists of glucose, indicating large amounts of glucan and much less glycoprotein than found in vegetative cell walls. The linkage analysis shows that 1,4-glucose linkages (30.7%) are more prevalent than 1,3-glucose linkages (25.4%), indicating that mixed β-1,3-/β-1,4-glucans are more abundant than β-1,3-glucans. If we assume the mixed β-1,3-/β-1,4-glucan is lichenin with a 2 to 1 ratio of β-1,4-linkages to β-1,3-linkages, then lichenin (approximately 45% of the carbohydrate) is approximately four times more abundant than β-1,3-glucan (approximately 10% of the carbohydrate) within the perithecia cell walls. As indicated below, some of the 1,3-glucan present in the perithecia cell walls is α-1,3-glucan, and the ratio of lichenin to β-1,3-glucan is likely greater than 4:1. The reduced levels of mannose and galactose in the perithecia cell walls suggests they contain much less glycoprotein-associated carbohydrate than found in the vegetative cell walls. When considering the perithecia cell walls, it is important to recognize that melanin is a major component of these cell walls. While melanin is not captured in our sugar analyses, it is an integral part of the *N. crassa* perithecia cell wall and clearly represents a major cell wall structural element [26].

The cell walls from the ascospores are markedly different from the other three cell walls examined. One of the most striking differences is the presence of large amounts of 4-linked glucosamines (17.2%) in the linkage analysis (Table 2). This represents a large amount of chitin or chitosan within the ascospore cell walls and far exceeds the amounts of chitin seen in the other cell wall types. This indicates that chitin is a major element in the ascospore cell walls and suggests that chitin helps provide structural integrity to the ascospore cell wall. The composition analysis (Table 1) and linkage data (Table 2) indicate that a second important difference is that ascospore cell walls contain more mannose and galactose than other cell walls. Since the mannose and galactose are largely derived from glycoprotein [16], the data suggests that the ascospore cell wall may contain more glycoprotein than other cell types. Consistent with having a major increase in chitin and glycoproteins, the ascospore cell wall contains a lower percentage of glucan (Table 2). Like the perithecia, ascospores are highly melanized, and this may have given some interference with the permethylation step in the linkage analysis (giving products with “extra linkages”). However, the data clearly shows that the ascospore cell walls contain more 1,4-glucose (22.2%) than 1,3-glucose (16.2%). This indicates that mixed β-1,3/β-1,4-glucans are more abundant than β-1,3-glucans. Like the situation observed for the perithecia, if we assume the mixed β-1,3/β-1,4-glucan to be lichenin with a 2 to 1 ratio of β-1,4 linkages to β-1,3-linkages, then lichenin (approximately 33% of the cell wall carbohydrate) is much more abundant than β-1,3-glucan (approximately 5% of the cell wall carbohydrate) in the ascospore cell walls. As with the perithecia, it is important to note that melanin is a major cell wall component in ascospore cell walls. In a study on melanin formation in *N. crassa,* we suggest the melanin granules encase the cell wall matrix to generate a structure reminiscent of reinforced concrete [26]. *N. crassa* ascospores are able to persist for decades in the environment [11] and the cell wall organization we have described for them provides some insights into how they might be able to survive. The presence of large amounts of chitin, an abundance of cell surface glycoproteins, and having the cell wall matrix encased within melanin would provide for a formidable cell wall structure.

### 3.3. Expression of the α-1,3-Glucan Synthase Across the N. crassa Life Cycle

**Figure 1:**
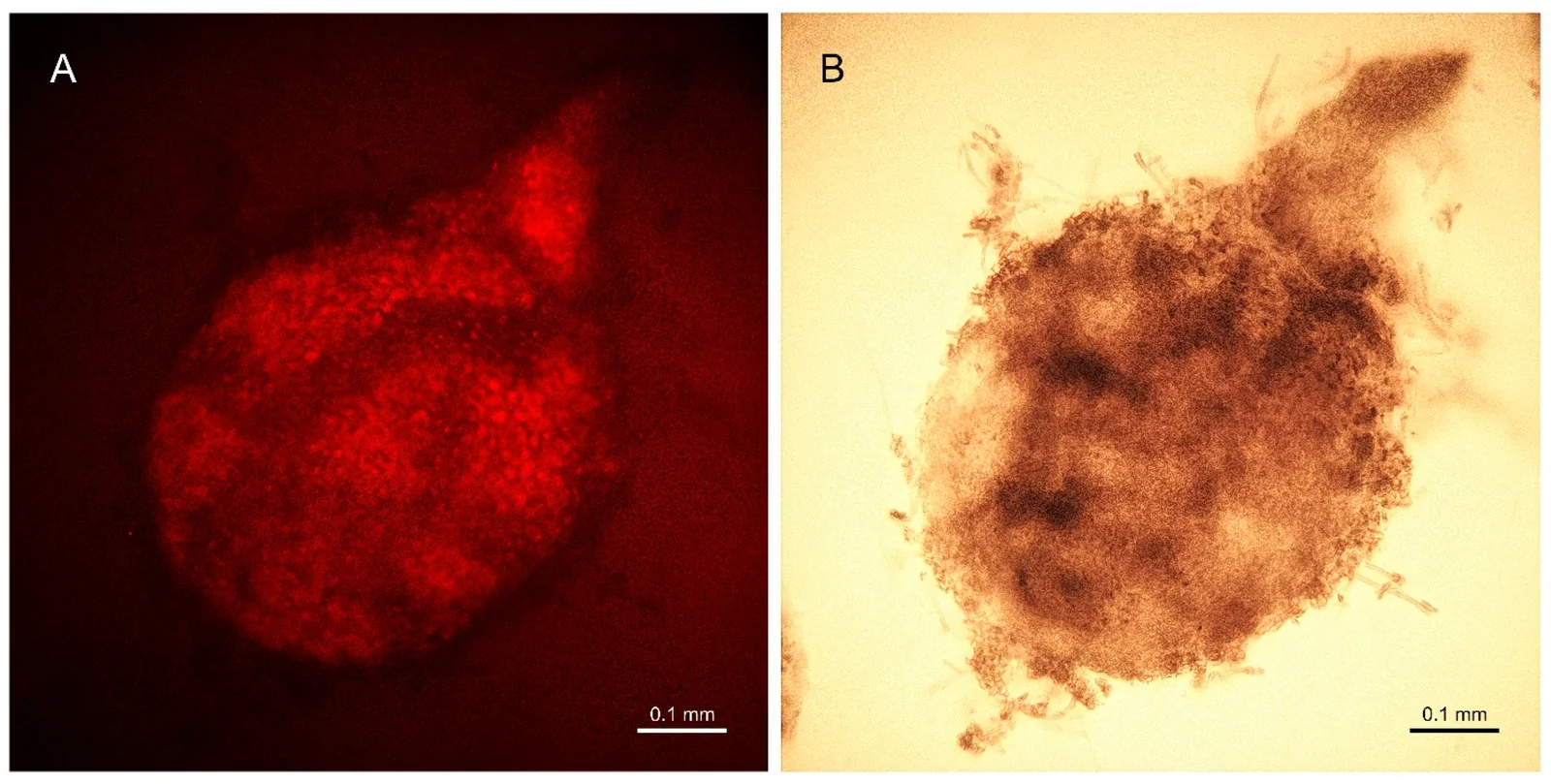
α-1,3-glucan synthase expression within the peridium. Fluorescent image (panel (**A**)) and Bright field image (panel (**B**)) of a perithecia squash from an isolate expressing RFP under the direction of the NCU08132/*ags-1* promoter. Note the expression of RFP within the peridium of the squashed perithecia.

We had previously examined the expression of a chimeric gene consisting of the promoter region from *ags-1*, the α-1,3-glucan synthase gene fused to the RFP coding region. We showed that *ags-1* is expressed in the conidia [10]. In our current study, we examined the expression of the chimeric gene during the time course of perithecia development and found that the gene was expressed in the perithecia. Figure 1 shows a representative image of expression of the chimeric gene within the developing perithecium. We did not see evidence of *ags-1* being expressed within the developing ascospores. We conclude that the 1,3-glucan identified within the perithecia includes some α-1,3-glucan as well as β-1,3-glucan.

## 4. Discussion

The structure and organization of fungal cell walls have been a major area of research interest. The cross-linked matrix of polysaccharides and glycoproteins is unique to fungi and has received attention as the target for anti-fungal agents. Most of the work on fungal cell walls has been carried out on vegetative hyphae cell walls. The major polysaccharides found in the characterized cell walls include chitin, β-1,3-glucan, β-1,6-glucan, α-1,3-glucan, and mixed β-1,3-/β-1,4-glucans [1,2]. In *N. crassa*, multiple cell wall glycoproteins have been identified, including GH16, GH17, GH18, GH72, and GH76 family enzymes, some of which have been shown to be capable of cross-linking the cell wall polysaccharides together to create a cell wall matrix [1,2,19,20,27]. These enzymes are found to be encoded by multigene families, and we identified members of these gene families in our previous hyphae and conidia cell wall proteomic analyses [14,15,28]. Many cell wall glycoproteins have been found to have a GPI-anchor (glycosylphosphatidylinositol anchor) at their carboxyl terminus, and several GPI-anchored proteins were identified in our work.

The purpose of the present study was to characterize how the cell wall composition and structure change throughout the fungal life cycle. We previously examined the composition and sugar linkages in the vegetative hyphae cell wall [12,13,15,16]. In this study, we characterized the composition and sugar linkages in cell walls from conidia (asexual spores), perithecia (female mating structure), and ascospores (sexual spores). Table 3 summarizes the differences we found in our research. Our data indicates that each of the four cell walls examined differs from the other three in glucan composition and glycoprotein content. The data is consistent with β-1,3-glucan and lichenin (mixed β-1,3-/β-1,4-glucan) being major cell wall polysaccharides in all of the cell walls, but the relative amounts of these polysaccharides differ significantly in the four types of cell walls. The synthases for chitin and β-1,3-glucan are known [1,4], but the synthase(s) responsible for producing lichenin has not been elucidated yet. The *N. crassa* genome encodes seven chitin synthases, two α-1,3-glucan synthases, and a single β-1,3-glucan synthase gene [29].

The vegetative hyphae cell wall contains β-1,3-glucan and lichenin, with β-1,3-glucan being approximately twice as prevalent as lichenin (ratio of 51% to 24%) (Table 3). Approximately 12% of the cell wall carbohydrate comes from the post-translational modification in glycoproteins, indicating that glycoprotein is a major component of hyphae cell walls. Our previous work showed that seventeen of the glycoproteins we identified in a proteomic analysis of hyphae cell wall proteins were not found in the conidia cell wall [14]. This suggests that some hyphae glycoproteins may be unique to the hyphae cell wall.

Our analysis of the conidia cell walls showed them to be significantly different from the hyphae cell walls. One major difference is a large decrease in the amount of glycoprotein and an increase in the cell wall glucan content. Our results suggest that the conidia cell walls have approximately 1/3 the amount of glycoprotein found in the hyphae cell walls (Table 3). Our previous work has shown the conidia cell wall contains α-1,3-glucan [10]. Table 3 shows approximately 70% of the glucan in the conidia cell wall is found in 1,3-linkages, but the analysis does not distinguish between β-1,3-glucan and α-1,3-glucan. The conidia cell walls contain about 15% lichenin, a value that is lower than in any of the other cell walls (Table 3). The linkage analysis (Table 2) shows that 3,6-linked glucose was among the major sugars identified (5% of the sugars). This suggests that the conidia cell walls may have a higher degree of cross-linking between the polysaccharides within the polysaccharide matrix. A similar observation of abundant crosslinking within conidia cell walls has been previously observed for *Trichoderma atroviride* [9]. In our previous proteomic analysis of the conidia cell wall, we identified nineteen cell wall glycoproteins that were found in the conidia cell wall and not found in the vegetative cell wall [14]. We concluded that the proteome of the conidia cell wall differs from that of the vegetative hyphae.

The cell wall analysis for the perithecia largely reflects the cell wall composition of the peridium, the major cell type within the female mating structure. We found the perithecia cell walls contained less glycoprotein and more glucan than the hyphae. We estimate that the perithecia cell walls contain approximately 1/3 the amount of glycoprotein found in the hyphae cell walls. Interestingly, mixed β-1,3-/β-1,4-glucan (lichenin) was the major polysaccharide present (45%) with lower amounts of 1,3-glucan (10%) being present. The 1,3-glucan present in the perithecia cell walls consists of β-1,3-glucans and α-1,3-glucans (Figure 1), a finding consistent with the RNAseq data from developing perithecia which shows expression of mRNAs from the GLS-1 gene (FKS1/NCU06871—β-1,3-glucan synthase) and the AGS-1 gene (NCU08132—α-1,3-glucan synthase) [30,31,32]. Unlike the hyphae and conidia cell walls, the perithecia cell walls were melanized. From our results, we conclude that the perithecia cell walls, with mixed β-1,3-/β-1,4-glucan (lichenin) as the major polysaccharide and a reduced amount of glycoprotein, are significantly different from the other cell walls examined.

We find the ascospore cell walls to be dramatically different from the other three cell walls. The most striking difference is the presence of large amounts of chitin and/or chitosan, which we estimate to represent 17% of the cell wall (Table 3). This is compared to the near absence of chitin in the hyphae and conidia cell walls and 3.6% chitin in the perithecia cell walls. We estimate that, with the sensitivity of our analyses, we would be able to detect chitin levels over 1% (Table 2). In addition to the peridium cells, the perithecia contain developing ascospores as a minor component [24]. Some of the chitin and glycoprotein-associated carbohydrates seen in the perithecia cell wall sample may have come from the developing ascospores. A second difference is that the ascospore cell walls contain less glucan than that found in the other cell walls (Table 3). The major glucan present was found to be lichenin (33%), with lower amounts of β-1,3-glucan (5%), and the lowest amount of 1,3-linked glucan from the four cell wall types. We also found the ascospore cell walls contained more glycoprotein-associated carbohydrates than the other cell walls. We estimate that approximately 17% of the cell wall sugars come from glycoprotein post-translational modifications. This indicates the presence of large amounts of glycoprotein within the ascospore cell walls. An increase in chitin and glycoproteins has also been seen for *Saccharomyces cerevisiae* ascospores [7,8]. The *Aspergillus fumigatus* ascospore cell wall has also been found to have high amounts of chitin [5]. In considering the ascospore cell walls, it is important to note that the ascospore cell walls are heavily melanized and the melanin content is not captured by our analyses. In an earlier study on the formation of melanin from DHN (1,8-dihydroxynaphthalene) in *N. crassa*, it has been suggested that cell wall melanin granules are formed “in situ” (within the cell wall space) and encase the cell wall glucan/glycoprotein matrix within the forming granules to generate a structure reminiscent of “reinforced concrete” [26]. Based on these observations, we would represent the ascospore cell wall as a formidable structure, with large amounts of chitin and a polysaccharide matrix with an abundance of glycoprotein encased within melanin.

In summary, we have examined the composition and glycoprotein content of cell walls from vegetative hyphae, conidia, perithecia, and ascospores. We found the four types of cell walls to differ from each other in the amounts and types of polysaccharides present and in the amounts of glycoprotein. The data shows the *N. crassa* cell wall to be a dynamic structure and it is dramatically altered as the fungus progresses from one stage of its life cycle to another.

## Figures and Tables

**Table 1:** Analysis of the neutral sugars present in vegetative, conidia, ascospore, and perithecium cell wall samples.

Sugar Residue	Ascospore Cell Wall	Perithecia Cell Wall	Vegetative Cell Wall	Conidia Cell Wall
Arabinose	0.6%	0.2%	0.0%	0.0%
Xylose	0.5%	0.2%	0.2 ± 0.2%	0.0%
Mannose	15.7%	3.9%	9.8 ± 2.7%	3.6%
Galactose	5.6%	2.0%	6.8 ± 2.7%	2.3%
Glucose	77.6%	93.7%	83.2 ± 5.6%	94.1%

The mole percentage for each of the sugar residues in the four types of cell walls is shown. The mean and standard deviation for three separate determinations of the vegetative cell wall are included.

**Table 2:** Glycosyl linkages in cell wall samples.

Sugar Residue	Ascospore Cell Wall	Perithecia Cell Wall	Vegetative Cell Wall	Conidia Cell Wall
t-Galf	1.3%	0.2%	2.9 ± 1.4%	0.0%
4/5-Galf	0.0%	0.0%	3.8 ± 2.9%	0.0%
t-Gal	0.0%	0.0%	0.2 ± 0.3%	0.0%
3-Gal	0.0%	0.0%	0.5 ± 0.6%	2.9%
Total Gal	1.3%	0.2%	7.4%	2.9%
t-Man	4.5%	0.3%	0.7 ± 0.8%	0.0%
2-Man	0.4%	0.9%	1.7 ± 2.1%	0.0%
3-Man	0.2%	0.2%	0.0%	2.9%
4-Man	54.0%	1.7%	0.0%	0.0%
6-Man	0.5%	0.0%	0.4 ± 0.6%	0.2%
2,3-Man	0.0%	0.0%	0.7 ± 0.5%	0.0%
2,6-Man	3.4%	1.0%	1.3 ± 1.7%	0.0%
2,3,4,6-Man	2.8%	0.0%	0.2 ± 0.1%	0.0%
Total Man	15.8	4.1%	5.0%	3.1%
t-Glc	9.2%	9.4%	3.3 ± 1.2%	2.3%
2-Glc	0.0%	0.0%	1.2 ± 1.6%	0.0%
3-Glc	16.2%	25.4%	58.4 ± 8.6%	75.7%
4-Glc	22.2%	30.7%	15.8 ± 4.7%	10.1%
6-Glc	1.1%	0.3%	0.6 ± 0.9%	0.0%
2,3-Glc	3.0%	3.1%	3.2 ± 1.1%	0.5%
2,4-Glc	0.5%	1.4%	0.0%	0.0%
3,4-Glc	2.7%	4.3%	1.9 ± 1.4%	0.0%
3,6-Glc	2.5%	3.2%	2.0 ± 0.6%	5.1%
4,6-Glc	0.7%	1.5%	0.2 ± 0.3%	0.0%
2,3,4-Glc	2.0%	4.9%	0.2 ± 0.3%	0.0%
3,4,6-Glc	0.8%	1.6%	0.0%	0.0%
2,3,4,6-Glc	4.8%	7.3%	0.2 ± 0.1%	0.0%
Total Glc	65.7%	91.6%	87.0%	93.7%
t-GlcNac	0.5%	0.0%	0.0%	0.0%
4-GlcNac	17.2%	3.6%	0.0%	0.0%
Total GlcNac	17.7%	3.6%	0.0%	0.0%

**Table 3:** Summary of cell wall components from different types of cell walls.

Cell Wall Component	Vegetative Cell Walls	Conidia Cell Walls	Perithecia Cell Walls	Ascospore Cell Walls
Chitin	nd	nd	3.6%	17%
1,3-glucan	51% (β-1,3-)	70% (β-1,3- & α-1,3-)	10% (β-1,3- & α-1,3-)	5% (β-1,3-)
Mixed glucan (lichenin)	24%	15%	45%	33%
Glycoprotein-associated carbohydrates	12%	6%	4%	17%
Melanin	0%	0%	+	+++

The approximate mole percentages of cell wall carbohydrate for each of the major cell wall components is given (data taken from Table 2). The designation of glycoprotein-associated carbohydrates is derived from the levels of mannose and galactose present. The nd designation indicates chitin was not detected in hyphae and conidia cell walls. The + and +++ designations for melanin denote the presence of melanin within the perithecia and ascospore cell walls.

## Data Availability

The original contributions presented in this study are included in the article. Further inquiries can be directed to the corresponding author.
